# Supplementary material for: Uncontrolled hypertension among hypertensive patients in Sub-Saharan Africa: A systematic review and meta-analysis
Source: PLoS One. 2024 Jun 13;19(6):e0301547. doi: 10.1371/journal.pone.0301547 (PMC11175416; doi:10.1371/journal.pone.0301547)
Supplement: S2 File — (DOCX) [file pone.0301547.s002.docx]

Supplemental Table 2: Quality assessment of the included studies using the Joanna Briggs Institute (JBI) quality appraisal criteria

| 1. **For cross-sectional studies** | | | | | | | | | | | | | | | | |  |
| --- | --- | --- | --- | --- | --- | --- | --- | --- | --- | --- | --- | --- | --- | --- | --- | --- | --- |
| **S/N** | | **Author [Year]** | **Criteria** | | | | | | | | | | | **Scores** | | **Overall quality** |  |
|  | |  | Clearly defined inclusion criteria | Describing the study settings participants | Valid &reliable exposure measurement | Objective &standard criteria for measurement | Identified confounder | | Strategies to deal with confounder | Valid & reliable outcome measurement | Appropriate statistical analysis | |  | |  | |  |
|  | | Abdissa[2022] | N | Y | Y | Y | N | | Y | N | Y | | 5 | | Low risk | |  |
|  | | Abdu[2017] | Y | Y | Y | Y | N | | Y | Y | Y | | 7 | | Low risk | |  |
|  | | Abegaz[2018] | Y | Y | Y | Y | N | | Y | Y | Y | | 7 | | Low risk | |  |
|  | | Abegaz[2022] | Y | Y | Y | Y | N | | Y | Y | Y | | 7 | | Low risk | |  |
|  | | Aberhe[2020] | Y | Y | Y | Y | N | | Y | Y | Y | | 7 | | Low risk | |  |
|  | | Antignac[2018] | N | Y | Y | Y | N | | Y | Y | Y | | 6 | | Low risk | |  |
|  | | Asgedom[2016] | Y | Y | Y | Y | N | | Y | Y | Y | | 7 | | Low risk | |  |
|  | | G/Mikael[2019] | Y | Y | Y | Y | N | | Y | Y | Y | | 7 | | Low risk | |  |
|  | | Douglas[2018] | Y | Y | Y | Y | N | | Y | Y | Y | | 7 | | Low risk | |  |
|  | | Fekadu[2022] | Y | Y | Y | Y | N | | Y | Y | Y | | 7 | | Low risk | |  |
|  | | Lemessa[2021] | Y | Y | Y | Y | N | | Y | Y | Y | | 7 | | Low risk | |  |
|  | | Lichisa[2014] | Y | Y | Y | Y | N | | Y | Y | Y | | 7 | | Low risk | |  |
|  | | Magara[2022] | Y | Y | Y | N | N | | Y | N | Y | | 5 | | Low risk | |  |
|  | | Maginga[2016] | N | Y | Y | Y | N | | Y | Y | Y | | 6 | | Low risk | |  |
|  | | Masilela[2020] | Y | Y | Y | Y | N | | Y | Y | Y | | 7 | | Low risk | |  |
|  | | Menanga[2016] | N | Y | Y | Y | N | | Y | Y | Y | | 6 | | Low risk | |  |
|  | | Muleta[2017] | N | Y | Y | Y | N | | Y | Y | Y | | 6 | | Low risk | |  |
|  | | Negash[2023] | Y | Y | Y | Y | N | | Y | Y | Y | | 7 | | Low risk | |  |
|  | | Omar[2018] | N | Y | Y | Y | N | | Y | N | Y | | 5 | | Low risk | |  |
|  | | Sheleme[2022] | Y | Y | Y | Y | N | | Y | Y | Y | | 7 | | Low risk | |  |
|  | | Solomon[2023] | Y | Y | Y | Y | N | | Y | Y | Y | | 7 | | Low risk | |  |
|  | | Tesfaye[2017] | N | Y | Y | Y | N | | Y | Y | Y | | 6 | | Low risk | |  |
|  | | Teshome[2018] | Y | Y | Y | Y | N | | Y | Y | Y | | 7 | | Low risk | |  |
|  | | Yazie[2018] | Y | Y | Y | Y | N | | Y | Y | Y | | 7 | | Low risk | |  |
|  | 1. **For cohort studies** | | | | | | | | | | | | | | | | |
|  | **S/N** | **Criteria** | | | | | | | **Anmut *et al* [2022]** | | | | **Berhe *et* al [2016]** | | | | |
|  | 1. 1. | Two groups are similar and recruited from the same population | | | | | | | Y | | | | Y | | | | |
| 1. 2. | Similar measurement of exposure both for exposed and unexposed groups | | | | | | | Y | | | | Y | | | | |  |
| 1. 3. | Valid and reliable measurement of exposure | | | | | | | Y | | | | Y | | | | |  |
| - 1. 4 | Identifying confounders | | | | | | | N | | | | N | | | | |  |
| 1. 5. | Strategies to deal with confounders | | | | | | | N | | | | N | | | | |  |
| 1. 6. | Groups are free of the outcomes at the beginning | | | | | | | Y | | | | Y | | | | |  |
| 1. 7. | Valid and reliable measurement of outcomes | | | | | | | Y | | | | Y | | | | |  |
| 1. 8. | Long enough follow-up time for the occurrence of outcomes | | | | | | | Y | | | | N | | | | |  |
| 1. 9. | Complete follow-up time | | | | | | | Y | | | | Y | | | | |  |
| 1. 10. | Strategies to address lost follow-up | | | | | | | Y | | | | Y | | | | |  |
| *Percentage (%) of ʺYesʺ* | | | | | | | | *8/10=80%* | | | | *7/10=70%* | | | | |  |

*Note: Y, yes; N, No*

Supplemental Table 3: Risk of bias assessment of the included studies

| **S/N** | **Author [Year]** | **Criteria** | | | | | | | | | | **Scores** | **Overall risk of bias** |
| --- | --- | --- | --- | --- | --- | --- | --- | --- | --- | --- | --- | --- | --- |
|  |  | **External validity** | | | | **Internal validity** | | | | | | |  |
|  |  | **Q1** | **Q2** | **Q3** | **Q4** | **Q5** | **Q6** | **Q7** | **Q8** | **Q9** | **Q10** | |  |
|  | Abdissa *et a*l [2022] | N | Y | Y | Y | Y | N | Y | Y | Y | Y | 8 | Low risk |
|  | Abdu *et a*l [2017] | N | Y | Y | Y | Y | Y | Y | Y | N | Y | 8 | Low risk |
|  | Abegaz *et a*l [2018] | N | Y | Y | Y | Y | Y | Y | Y | N | Y | 8 | Low risk |
|  | Abegaz *et a*l [2022] | N | Y | N | Y | Y | Y | Y | Y | Y | Y | 8 | Low risk |
|  | Aberhe *et a*l [2020] | N | Y | Y | Y | Y | Y | Y | Y | N | Y | 8 | Low risk |
|  | Anmut *et a*l [2022] | N | Y | Y | Y | N | Y | Y | Y | Y | Y | 8 | Low risk |
|  | Antignac *et a*l [2018] | N | Y | Y | Y | Y | N | Y | Y | Y | Y | 8 | Low risk |
|  | Asgedom *et a*l [2016] | N | Y | Y | Y | Y | Y | Y | Y | N | Y | 8 | Low risk |
|  | Berhe *et a*l [2016] | N | Y | Y | Y | N | Y | Y | Y | Y | Y | 8 | Low risk |
|  | G/Michael *et a*l [2019] | N | Y | Y | Y | Y | Y | Y | Y | N | Y | 8 | Low risk |
|  | Douglas *et a*l [2018] | N | Y | Y | Y | Y | Y | Y | Y | Y | N | 8 | Low risk |
|  | Fekadu *et a*l [2022] | N | Y | Y | Y | Y | Y | Y | Y | N | Y | 8 | Low risk |
|  | Lemessa *et a*l [2021] | N | Y | Y | Y | Y | Y | Y | Y | N | Y | 8 | Low risk |
|  | Lichisa *et a*l [2014] | N | Y | Y | Y | Y | Y | Y | Y | N | Y | 8 | Low risk |
|  | Magara *et a*l [2022] | N | Y | Y | Y | Y | N | N | Y | Y | Y | 7 | Low risk |
|  | Maginga *et a*l [2016] | N | Y | Y | Y | Y | N | Y | Y | N | Y | 7 | Low risk |
|  | Masilela *et a*l [2020] | N | Y | Y | Y | Y | Y | Y | Y | N | Y | 8 | Low risk |
|  | Menanga *et a*l [2016] | N | Y | Y | Y | Y | N | Y | Y | Y | Y | 8 | Low risk |
|  | Muleta *et a*l [2017] | N | Y | Y | N | Y | N | Y | Y | Y | Y | 7 | Low risk |
|  | Negash *et a*l [2023] | N | Y | Y | Y | Y | Y | Y | Y | N | Y | 8 | Low risk |
|  | Omar *et a*l [2018] | N | Y | Y | Y | Y | N | N | Y | Y | Y | 7 | Low risk |
|  | Sheleme *et a*l [2022] | N | Y | Y | Y | Y | Y | Y | Y | Y | N | 8 | Low risk |
|  | Solomon *et a*l [2023] | N | Y | Y | Y | Y | Y | Y | Y | N | Y | 8 | Low risk |
|  | Tesfaye *et a*l [2017] | N | Y | Y | Y | Y | N | Y | Y | Y | Y | 8 | Low risk |
|  | Teshome *et a*l [2018] | N | Y | Y | Y | Y | Y | Y | Y | N | Y | 8 | Low risk |
|  | Yazie *et a*l [2018] | N | Y | N | Y | Y | Y | Y | Y | Y | Y | 8 | Low risk |

Note: Y, Yes; N, No; Q1, Representatives of the target population; Q2, Representativeness of the sampling frame; Q3, Random sampling or census; Q4, Minimal response bias; Q5, Data were collected directly; Q6, Acceptable case definition used in the study; Q7, Valid and reliable measurement; Q8, The same mode of data collection for all study subject; Q9, Appropriate length of prevalence period for parameter of interest and Q10, Appropriate numerators and denominators of interest.
