# Supplementary material for: Uncontrolled hypertension among hypertensive patients in Sub-Saharan Africa: A systematic review and meta-analysis
Source: PLoS One. 2024 Jun 13;19(6):e0301547. doi: 10.1371/journal.pone.0301547 (PMC11175416; doi:10.1371/journal.pone.0301547)
Supplement: Attachment — Submitted filename: Response to Reviewers.docx [file pone.0301547.s003.docx]

**Dear Editors and Reviewers:**

We sincerely appreciate the valuable comments and suggestions you raised. The thorough review helped immensely in the shaping of the manuscript. The comments and suggestions have been closely followed and revisions have been made accordingly. The following are the questions extracted from the Editors and Reviewers’ comments along with our summarized responses. Thank you very much for your constructive comments. We tried to inculcate your comments and questions as described below. The changes will be attached with

Title: Uncontrolled hypertension and its predictors among hypertensive patients in Ethiopia and selected Sub-Saharan African countries: Systematic review and Meta-analysis.

**Authors:**

TM: [*tigabumunye21@gmail.com*](mailto:tigabumunye21@gmail.com)

AK: [amarekassaw2009@gmail.com](mailto:amarekassaw2009@gmail.com)

AS: [amaresimegn99@gmail.com](mailto:amaresimegn99@gmail.com)

GN: [gedefayen@gmail.com](mailto:gedefayen@gmail.com)

SA: [sintie579@gmail.com](mailto:sintie579@gmail.com)

YT: [tesfahunyohannes08@gmail.com](mailto:tesfahunyohannes08@gmail.com)

SD: [solomondemis@gmail.com](mailto:solomondemis@gmail.com)

DK: [demewozk@yahoo.com](mailto:demewozk@yahoo.com)

SZ: [shegawzn@gmail.com](mailto:shegawzn@gmail.com)

WN: [workunecho@gmail.com](mailto:workunecho@gmail.com)

**Editor Comment #01:** Please ensure that your manuscript meets PLOS ONE's style requirements, including those for file naming. The PLOS ONE style templates can be found at

<https://journals.plos.org/plosone/s/file?id=wjVg/PLOSOne_formatting_sample_main_body.pdf>  and <https://journals.plos.org/plosone/s/file?id=ba62/PLOSOne_formatting_sample_title_authors_affiliations.pdf>

**Authors’ response:** Recognizing your comment, we have looked at the PLOS ONE style templates using the given link and we have ensured that our manuscript meets PLOS ONE's style requirements, including those for file naming. The requested corrections have been included throughout the revised version manuscript.

**Editor Comment #02:** We noticed you have some minor occurrence of overlapping text with the following previous publication(s), which needs to be addressed:

- <https://doi.org/10.1111/tmi.13684>

- <https://doi.org/10.1371/journal.pone.0220710>

- <https://doi.org/10.2147/IBPC.S245068>

**Authors’ response:** We are convinced of this comment, and we have revised these overlapping texts in the manuscript accordingly.

**Editor Comment #03:** In your revision ensure you cite all your sources (including your own works), and quote or rephrase any duplicated text outside the methods section.

**Authors’ response:** Accepting your valuable comment, we have revised the duplicated texts outside the method section of the manuscript.

**Editor Comment #04:** Please include a separate caption for each figure in your manuscript.

**Authors’ response:** Recognizing your comment, we included a separate caption for each figure in the manuscript.

**Reviewer # 1:**

**Reviewer # 1 comment and suggestion #01:** Title: Uncontrolled Hypertension and Its Predictors among Hypertensive Patients in Sub-Saharan Africa: Systematic Review and Meta-Analysis.
In this systematic review and meta-analysis, Aytenew et al aimed to determine the pooled burden of uncontrolled hypertension and identify its predictors. This is a commendable effort. However, there are several limitations, and some were not identified in the manuscript.

Firstly, why restrict the search to studies published between 2014 and 2023 in SSA? The reason for this should be explained in the discussion, because several important studies were omitted. In a previous meta-analysis on a similar subject, Addo et al included 37 studies published between 1975 and 2006 (Addo J, Smeeth L, Leon DA. Hypertension in Sub-Saharan Africa: a systematic review. Hypertension. 2007 Dec;50(6):1012-8. doi: 10.1161/HYPERTENSIONAHA.107.093336. Epub 2007 Oct 22.), which I believe would have enriched the present meta-analysis.

**Authors’ response**: Thank you for your critical view of our manuscript! Today, the diagnostic modalities, treatment protocols, burden of chronic illnesses including hypertension and the living styles of the community are changed through time. Therefore, the findings of the 37 studies published between 1975 and 2006 might not represent the current situations (they are outdated). We have searched extensively all available primary studies published after this review across the region, unfortunately, the searched studies laid between the year 2014 and 2023. So, when we said “the search was restricted to studies published between 2014 and 2023”, to mean that unfortunately, the searched studies laid between 2014 and 2023 in SSA.

**Reviewer #1 comment and suggestion #02:** Secondly and importantly, 19/24 studies in the present meta-analysis were from Ethiopia (i.e., two-thirds of the studied population). This would make the title of the manuscript inappropriate. Thus, the investigators may consider expanding the meta-analysis to span across more years and include studies from other countries. Otherwise, the title should be modified to show that most of the data is from Ethiopia, for example "Uncontrolled Hypertension and Its Predictors among Hypertensive Patients in Ethiopia and selected Sub-Saharan African countries: Systematic Review and Meta-Analysis".

**Authors’ response:** We have accepted the given comment fully. Instead of including studies conducted outside SSA (aimed to determine the pooled prevalence of uncontrolled hypertension in SSA), we have fully accepted your feedback to modify the title, and we have revised the title directly based on the given direction.
