# Supplementary material for: Uncontrolled hypertension among hypertensive patients in Sub-Saharan Africa: A systematic review and meta-analysis
Source: PLoS One. 2024 Jun 13;19(6):e0301547. doi: 10.1371/journal.pone.0301547 (PMC11175416; doi:10.1371/journal.pone.0301547)
Supplement: Attachment — Submitted filename: Response to Reviewers.docx [file pone.0301547.s004.docx]

**Dear Editors and Reviewers:**

We sincerely appreciate the valuable comments and suggestions you raised. The thorough review helped immensely in the shaping of the manuscript. The comments and suggestions have been closely followed and revisions have been made accordingly. The following are the questions extracted from the Editors and Reviewers’ comments along with our summarized responses. Thank you very much for your constructive comments. We tried to inculcate your comments and questions as described below. The changes will be attached with

Title: Uncontrolled hypertension among hypertensive patients in Sub-Saharan Africa: Systematic review and Meta-analysis.

**Authors:**

TM: [*tigabumunye21@gmail.com*](mailto:tigabumunye21@gmail.com)

AK: [amarekassaw2009@gmail.com](mailto:amarekassaw2009@gmail.com)

AS: [amaresimegn99@gmail.com](mailto:amaresimegn99@gmail.com)

GN: [gedefayen@gmail.com](mailto:gedefayen@gmail.com)

SA: [sintie579@gmail.com](mailto:sintie579@gmail.com)

YT: [tesfahunyohannes08@gmail.com](mailto:tesfahunyohannes08@gmail.com)

SD: [solomondemis@gmail.com](mailto:solomondemis@gmail.com)

DK: [demewozk@yahoo.com](mailto:demewozk@yahoo.com)

SZ: [shegawzn@gmail.com](mailto:shegawzn@gmail.com)

WN: [workunecho@gmail.com](mailto:workunecho@gmail.com)

**Reviewer #1:**

**Reviewer #1 comment and suggestion #01:** I still believe that if the authors can remove "Ethiopia" from their search terms, they will get several additional articles to include in the analysis. Including "Ethiopia" in the search terms will surely introduce bias in the result. They can take a look at the article cited below to see that the use of appropriate search terms could reveal a more robust result:
Ataklte F, Erqou S, Kaptoge S, Taye B, Echouffo-Tcheugui JB, Kengne AP. Burden of undiagnosed hypertension in Sub-Saharan Africa: A systematic review and meta-analysis. Hypertension. 2015 Feb; 65(2):291-8. Doi: 10.1161/HYPERTENSIONAHA.114.04394. Epub 2014 Nov 10. PMID: 25385758.

**Authors’ response**: Thank you for your critical view of our manuscript! We tried to search articles extensively using different MeSH terms, even by removing "Ethiopia" across Sub-Saharan African countries. During the search process, we have already searched the above cited article (Burden of undiagnosed hypertension in Sub-Saharan Africa: A systematic review and meta-analysis. Hypertension, 2015 Feb; 65(2):291-8), but it was not in line with our title (Uncontrolled hypertension…).

**Reviewer #2:**

**Reviewer #2 comment and suggestion #01:** A recently published systematic review (SR) covers the same subject matter and research inquiry. If the authors intend to examine the prevalence of uncontrolled hypertension in low- and middle-income countries, I might consider reviewing a manuscript. Alternatively, to prevent duplicating published work, I recommend the authors to conduct a risk/etiology SR concerning risk factors for uncontrolled hypertension in Ethiopia. Click the link below to see the similar articles published recently: 10.1186/s12872-020-01414-3
Uncontrolled hypertension in Ethiopia: A systematic review and meta-analysis of institution-based observational studies | BMC Cardiovascular Disorders | Full Text ([biomedcentral.com](http://biomedcentral.com/)).

**Authors’ response:** Definitely! Our primary objective was to estimate the pooled prevalence of uncontrolled hypertension among hypertensive patients in Sub-Saharan Africa, and we have also cited the above reference on reference number 14 in the manuscript.

**Reviewer #3:**

**Reviewer #3 comment and suggestion #01:** Table 1 better present population characteristics, for examples age, race, sex, and more.

**Authors’ response:** Thank you for your valuable comment! We have revised the table based on the given direction, but the age of the target population was already operationalized in the manuscript (Age ≥18 years).

**Reviewer #3 comment and suggestion #02:** Table 1 does not correctly display the studys’ corresponding references. : Abegaz TM et al [2022] cannot be found in the reference list. There is a typo in "Abdissa." Animut's reference lists the year as "2018," but Table 1 indicates "2022."

**Authors’ response:** Thank you for your critical view and constructive comment! We have revised the studys’ corresponding references in Table 1 accordingly.

**Reviewer #3 comment and suggestion #03:** It is better to add a reference index number to each study.

**Authors’ response:** Thank you for your valuable comment! We have put a reference index number to each study in the table.

**Reviewer #3 comment and suggestion #04:** Several studies were conducted at UoGH around same time. Are they derived from the same patient cohort?

**Authors’ response:** Thank you for your valuable comment! These studies were carried out at UoGH on different patient cohort within some time variation ranging from 2017 to 2022.

**Reviewer #3 comment and suggestion #05:** Line 256, the selection of the random-effects model should be based on the research question and study design. It is not selected for assessing the influence of a single primary study on the overall meta-analysis.

**Authors’ response:** Accepting your valuable comment, we have revised it

**Reviewer #3 comment and suggestion #06:** Line 255 sensitivity analysis:
The funnel plot is not a form of sensitivity analysis. The paragraph is not clearly written. Figure 3 (not Figure 4) does not appear to be symmetric.

**Authors’ response:** Thank you for your valuable comment! We have revised this paragraph. But the sensitivity analysis was located on fig 4 not fig 3, because fig 3 was already located above this section/paragraph stating publication bias.

**Reviewer #3 comment and suggestion #07:** Subgroup analyses by sample sizes or by the year 2020 are irrelevant and arbitrary. It would be more appropriate to perform subgroup analyses based on regions (such as Ethiopia, SE Ethiopia, NE Ethiopia) or similar age groups and other relevant characteristics.

**Authors’ response:** Accepting your valuable comment, we have done the subgroup analysis using the study setting/area.

**Reviewer #3 comment and suggestion #08:** Line 276 and predictors:
This section is not clearly written. For cross-sectional studies, the relationship is more likely to represent an association rather than a prediction. It is unclear how the adjusted odds ratios were calculated. Were these calculated from pooled analysis? Did the studies adjust for the same covariates in logistic regression? If not, the pooled results may be influenced by bias.

**Authors’ response:** Thank you for your constructive comments! Yes! For cross-sectional studies, the relationship is more of an association not a prediction. Therefore, we have revised this section based on the given direction. And the adjusted odds ratios were calculated from the pooled analysis. The studies were also adjusted for the same covariates in logistic regression.
