# Supplementary material for: Uncontrolled hypertension among hypertensive patients in Sub-Saharan Africa: A systematic review and meta-analysis
Source: PLoS One. 2024 Jun 13;19(6):e0301547. doi: 10.1371/journal.pone.0301547 (PMC11175416; doi:10.1371/journal.pone.0301547)
Supplement: Attachment — Submitted filename: Response to Reviewers.docx [file pone.0301547.s005.docx]

**Dear Editors and Reviewers:**

We sincerely appreciate the valuable comments and suggestions you raised. The thorough review helped immensely in the shaping of the manuscript. The comments and suggestions have been closely followed and revisions have been made accordingly. The following are the questions extracted from the Editors and Reviewers’ comments along with our summarized responses. Thank you very much for your constructive comments. We tried to inculcate your comments and questions as described below. The changes will be attached with

Title: Uncontrolled hypertension among hypertensive patients in Sub-Saharan Africa: A systematic review and meta-analysis.

**Authors:**

TM: [*tigabumunye21@gmail.com*](mailto:tigabumunye21@gmail.com)

AK: [amarekassaw2009@gmail.com](mailto:amarekassaw2009@gmail.com)

AS: [amaresimegn99@gmail.com](mailto:amaresimegn99@gmail.com)

GN: [gedefayen@gmail.com](mailto:gedefayen@gmail.com)

SA: [sintie579@gmail.com](mailto:sintie579@gmail.com)

YT: [tesfahunyohannes08@gmail.com](mailto:tesfahunyohannes08@gmail.com)

SD: [solomondemis@gmail.com](mailto:solomondemis@gmail.com)

DK: [demewozk@yahoo.com](mailto:demewozk@yahoo.com)

SZ: [shegawzn@gmail.com](mailto:shegawzn@gmail.com)

WN: [workunecho@gmail.com](mailto:workunecho@gmail.com)

**Editor Comment #01:**

Please review your reference list to ensure that it is complete and correct. If you have cited papers that have been retracted, please include the rationale for doing so in the manuscript text, or remove these references and replace them with relevant current references. Any changes to the reference list should be mentioned in the rebuttal letter that accompanies your revised manuscript. If you need to cite a retracted article, indicate the article’s retracted status in the References list and also include a citation and full reference for the retraction notice.

**Authors’ response**: Thank you for your critical view of our manuscript! We have reviewed and revised our reference list intensively.

**Editor Comment #02:**

Please provide a detailed explanation of the search strategy in the revised manuscript. As mentioned in the response letter, it appears that you revised the search strategy using various MeSH terms and removing ‘Ethiopia’ to conduct an extensive search across Sub-Saharan African countries.

However, the results section indicates that the number of studies captured in the new search strategy for sub-Saharan Africa does not seem accurate. The new strategy did not capture additional studies, as evidenced in the results section where 19 out of 26 studies are from Ethiopia. This suggests that the key strategy may not have been updated correctly.

**Authors’ response:** Thank you for your valuable comment. We have provided a detailed explanation of the search strategy to get additional primary studies across Sub-Saharan African countries using various MeSH terms and removing ‘Ethiopia’ in the revised manuscript. However, we couldn’t get any more for this review.

**Editor Comment #03:**

While revising your submission, please upload your figure files to the Preflight Analysis and Conversion Engine (PACE) digital diagnostic tool, <https://pacev2.apexcovantage.com/>. PACE helps ensure that figures meet PLOS requirements. To use PACE, you must first register as a user. Registration is free. Then, login and navigate to the UPLOAD tab, where you will find detailed instructions on how to use the tool.

**Authors’ response:** Thank you! We have used the PACE tool to make our figures suitable for PLOS requirements.
